# Proteomic Identification of Novel Differentiation Plasma Protein Markers in Hypobaric Hypoxia-Induced Rat Model

**DOI:** 10.1371/journal.pone.0098027

**Published:** 2014-05-19

**Authors:** Yasmin Ahmad, Narendra K. Sharma, Mohammad Faiz Ahmad, Manish Sharma, Iti Garg, Kalpana Bhargava

**Affiliations:** 1 Peptide and Proteomics Division, DIPAS, DRDO, Ministry of Defence, Delhi, India; 2 Department of Chemistry, Jamia Millia Islamia, New Delhi, India; 3 Department of Genomics, DIPAS, DRDO, Ministry of Defence, Delhi, India; Duke University Medical Center, United States of America

## Abstract

**Background:**

Hypobaric hypoxia causes complex changes in the expression of genes, including stress related genes and corresponding proteins that are necessary to maintain homeostasis. Whereas most prior studies focused on single proteins, newer methods allowing the simultaneous study of many proteins could lead to a better understanding of complex and dynamic changes that occur during the hypobaric hypoxia.

**Methods:**

In this study we investigated the temporal plasma protein alterations of rat induced by hypobaric hypoxia at a simulated altitude of 7620 m (25,000 ft, 282 mm Hg) in a hypobaric chamber. Total plasma proteins collected at different time points (0, 6, 12 and 24 h), separated by two-dimensional electrophoresis (2-DE) and identified using matrix assisted laser desorption ionization time of flight (MALDI-TOF/TOF). Biological processes that were enriched in the plasma proteins during hypobaric hypoxia were identified using Gene Ontology (GO) analysis. According to their properties and obvious alterations during hypobaric hypoxia, changes of plasma concentrations of Ttr, Prdx-2, Gpx -3, Apo A-I, Hp, Apo-E, Fetub and Nme were selected to be validated by Western blot analysis.

**Results:**

Bioinformatics analysis of 25 differentially expressed proteins showed that 23 had corresponding candidates in the database. The expression patterns of the eight selected proteins observed by Western blot were in agreement with 2-DE results, thus confirming the reliability of the proteomic analysis. Most of the proteins identified are related to cellular defense mechanisms involving anti-inflammatory and antioxidant activity. Their presence reflects the consequence of serial cascades initiated by hypobaric hypoxia.

**Conclusion/Significance:**

This study provides information about the plasma proteome changes induced in response to hypobaric hypoxia and thus identification of the candidate proteins which can act as novel biomarkers.

## Introduction

High altitude is characterized as a region of low barometric pressure (hypobaric), low partial pressure of oxygen (hypoxia), severe cold and increase in ultraviolet radiation. High altitude poses several operational problems to the sojourners, soldiers and mountaineers not only during their initial days of induction to the hypoxic environment but also followed prolonged residency. With an increase in altitude, atmospheric pressure and the partial pressure of oxygen decrease rapidly leading to decreased O_2_ availability. This thus results in a condition termed as hypobaric hypoxia which stresses biological systems because of non-availability of steady uninterrupted supply of oxygen for mitochondrial metabolism. The cellular responses to hypobaric hypoxia are complex and characterized by alteration in the expression of a number of genes, including stress related genes and corresponding proteins that are necessary to maintain homeostasis [1]. Genes and their products (mRNA and proteins) that respond to hypobaric hypoxia have a great potential to serve as indicators of hypoxic stress, including enzymes of the glycolytic pathway, (which increase anaerobic ATP production), glucose transporters, enzymes involved in amino acid metabolism and gluconeogenesis, (which maintain blood glucose levels) [2], and heat shock proteins (which are involved in protein stability and folding) [3]. In general, genes that encode proteins involved in energy production, protein synthesis and degradation, lipid and carbohydrate metabolism, locomotion and contraction, and antioxidant defense are also the potential biomarkers of hypoxic stress [2]. Transcript levels of genes encoding specific proteins which can deal with perceived stressors are usually the first measurable biomarkers that can be assessed. However, examining gene expression alterations by itself does not give a complete picture as it is also essential to quantify the protein activity to ascertain that altered gene expression also results in altered protein levels. Changes in specific gene expression levels as well as the protein levels are excellent indicators that the organism has mobilized metabolic pathways in response to a specific stimulus.

A broader understanding of hypoxia-induced alterations in cellular or organ function could be better achieved from a combined knowledge derived from the concerted application of genomic and proteomics approaches. Although genomic changes during hypoxia have been extensively investigated, hypoxia-induced changes in the proteome of mammalian cells are only in the early phase of investigation. So far, a large number of studies have focused on the influence of hypoxia on the expression and posttranslational modification of a single protein of interest or a subset of functionally related proteins; however, very few reports have really examined proteome-wide alteration during hypoxia, with most focussing on cell lines [4]–[6].

The aim of the present study was to explore changes in the plasma proteome of rat exposed to hypobaric hypoxia at different time points (0 h, 6 h, 12 h and 24 hrs) and the levels of a specific protein of interest following hypobaric hypoxia have been measured by using proteomics tools. Plasma has the advantage over cells or tissue because it can be collected in a relatively non-invasive manner and has an immense diagnostic potential [7]. Moreover, all the protein components are readily accessible in a single compartment without requiring additional extraction procedures. Additionally, its constituents reflect closely the physiological and pathological alterations due to any stress condition [8]. Advances in proteomic techniques have facilitated the investigation of global changes in plasma proteomes. A variety of complementary procedures for the global analysis of protein expression have been described. These include two-dimensional gel electrophoresis (2-DE) coupled with mass spectrometry (MS) [9], multidimensional chromatography coupled with tandem MS [10], and chip technologies coupled with either antigens [11] or antibodies [12]. Among them, 2-DE coupled with matrix-assisted laser desorption/ionization MS is a prominent method for the identification of the proteins as well as for quantification of changes in protein expression [9], [13]. This proteomics analysis can provide information pertaining to compensatory changes occurring at the level of protein expression by altering antioxidant/oxidant status as a consequence of prior transcriptional and translational alterations in response to hypoxia and environmental perturbations.

## Results

**Figure 1:**
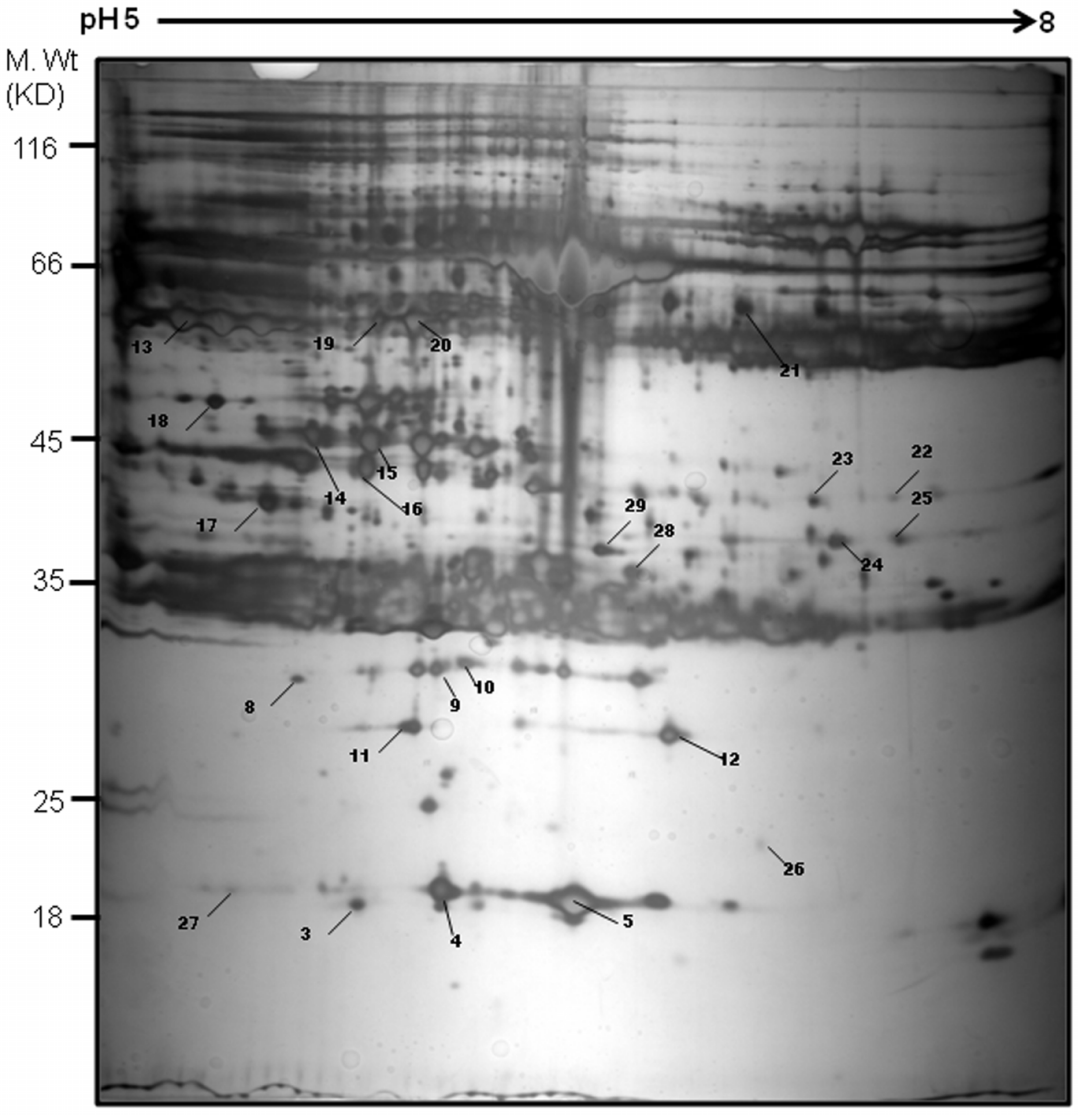
A representative 2D gel of plasma proteins from hypobaric hypoxia treated rat, with a pH range from 5–8. Distribution of differentially expressed protein spots and each spot number relates to data shown in Table 1.

**Figure 2:**
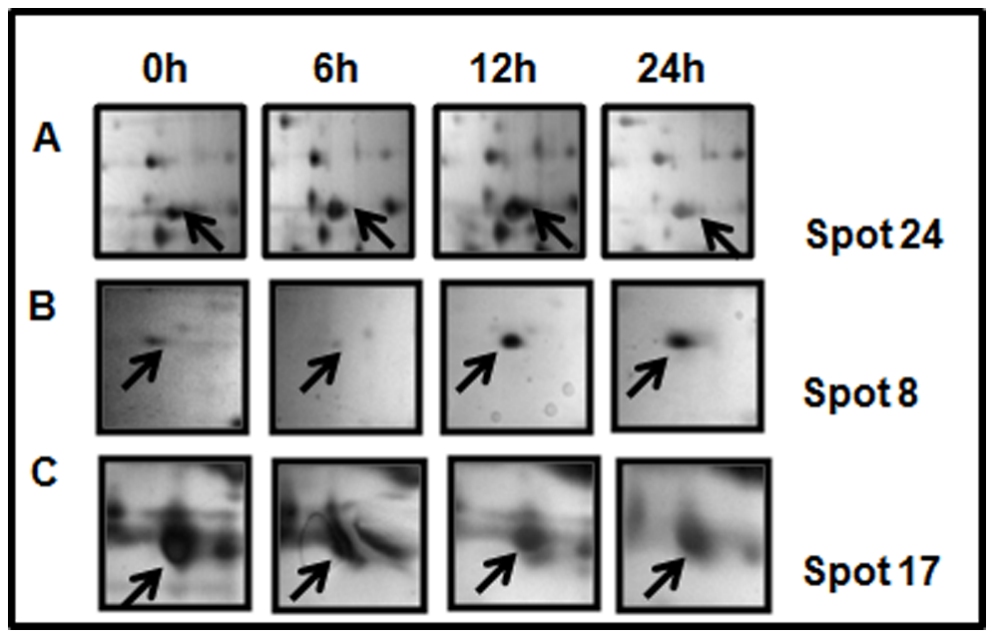
Magnified comparison maps of (A) spot 24, (B) spot 8 and (C) spot 17 in the 2-DE patterns with samples obtained at different time points after exposure to acute hypobaric hypoxia. Spot 24 was expressed in the control total plasma proteins but disappeared after acute hypobaric hypoxia. Spot 8 had low expression in the control group but its expression increased at each time point after acute hypobaric hypoxia. Spot 17 had high expression in the control group, but its expression decreased steadily after acute hypobaric hypoxia.

**Figure 3:**
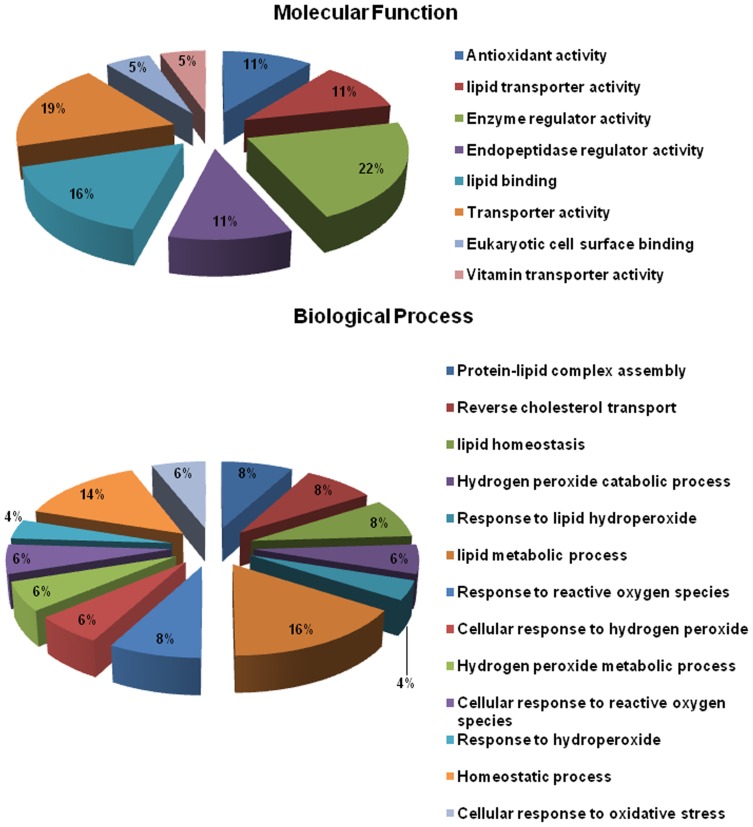
Gene ontology annotations of the proteins identified by MALDI-TOF/MS. Results were obtained using Blast2GO annotation. The distributions of identified proteins according to their (A) molecular functions and (B) biological processes are shown.

The protein expression profile in the plasma of hypoxia treated and control rats were obtained by two-dimensional electrophoresis (2-DE) with linear IPG strips of 17 cm, and pH range from 5–8. Proteins were resolved according to their isoelectric point (pI) in the first dimension and their molecular weight (Mw) in the second dimension. Gel images and representative 2-DE maps were unambiguously matched by the progenesis 2D-software, and displayed well-resolved and reproducible profiles for hypoxia treated and control rats. Approximately 500–800 protein spots were detected by silver staining in a single 2-DE gel and were distributed across the pI range 5–8, with molecular masses between 10000 and >130000 Da (Figure 1). On a 2-DE gel, one protein may be represented as a single spot or may be present as multiple spots, due to changes in molecular mass caused by post translational modification of the primary protein product or as a result of protein processing. Such modifications may also result in alterations of pI or conformation of a protein and consequently cause a shift in its position on the 2-D gel, thus resulting in change of spot intensity. Spots can also be considered in terms of spot families, representing the multiple spots within a 2-DE protein pattern created from a single primary protein. The quantity of each spot in a gel was normalized as a percentage of the total quantity of all the spots in the gel. In comparison with 2-DE patterns, protein spots with significant changes (p<0.05, one–way ANOVA) in a consistent direction (increase or decrease) were judged as deregulated and were chosen for identification. These differentially expressed plasma proteins had three types of expression patterns: (1) expressed in the control but not detected after exposure to acute hypoxia, (2) expressed slowly in the control group, but increased after exposure to acute hypoxia, irrespective of the time of exposure, and, (3) expressed highly in the control group, but decreased steadily after acute hypoxia. Figure 2 shows a magnified comparison of the patterns of spot 24 (Figure 2A), spot 8 (Figure 2B), and spot 17 (Figure 2C) which exemplify the main types of time-course for concentration levels after hypoxia. 25 differential protein spots were detected, of which 16 spots were up-regulated (3, 4, 5, 8, 9, 10, 13, 14, 15, 16, 19, 21, 22, 23, 26 and 27), while 9 spots were down-regulated (11, 12, 17, 18, 20, 24, 25, 28 and 29). In the present study, we considered a ≥20% change as significant and sufficient to include all the proteins for which even a 20% change in their expression levels (even at low fold change) is likely to have a functional relevance. Out of the 25 significantly deregulated spots, 23 were successfully identified by MALDI-TOF/TOF with PMF and MS/MS analysis followed by database searching (Figure 1, Table 1). Table 1 summarizes the identification information for these identified protein spots including protein name, accession number, molecular weight, pI value, and protein function. For spots 25 and 27, corresponding proteins in the database could not be found even after using the PMF and MS/MS searching. These may be novel proteins or else they may be small fragments of some proteins as can be said based on their molecular weight (≥15 kDa). If they would have been only smaller fragments, the PMF information for these two spots could be limited resulting in the failure of detection of the corresponding proteins in the database. The differentially expressed proteins listed here represent a wide range of biological categories. Proteins related to cellular defense mechanisms involving anti-inflammatory and antioxidant activity were the most common. When organized according to their molecular functions, 22% of the identified proteins correspond to those involved in binding and enzymatic regulatory activity, 11% involved in antioxidant activity and 19% in transporter activity (Figure 3). We also categorized proteins according to their biological processes; most abundant groups of proteins correspond to those involved in homeostatic process (16%), cellular response to oxidative stress (6%), cellular response to reactive oxygen species (8%) and lipid metabolic process (14%). Regarding their intensity rates and cellular functions in the plasma of hypoxia treated rats, transthyretin (Ttr), peroxiredoxin-2 (Prdx-2), glutathione peroxidase-3 (Gpx-3), Apolipoprotein A-I (ApoA-1), haptoglobin (Hp), Apolipoprotein-E (Apo-E), fetuin–B (Fetub) and Nucleoside diphosphate kinase B (Nme) were validated by Western blotting.

**Table 1:** List of differentially expressed rat plasma proteins after treated with hypobaric hypoxia, identified by MALDI-MS/MS.

Spot No.	SwissProtAccession no.	MascotScore	Protein Name	TheoreticalpI/MW	ObservedpI/MW	Fold Change			Function
						6 h	12 h	24 h	
4	P02767	198	Transthyretin	5.77/15	5.99/14			1.31**^b^**	Acute phase protein
8	P35704	129	Peroxiredoxin-2	5.34/22	5.52/22	0.77**^c^**		1.42^a^	Response to oxidative stress, Antioxidant
9	P23764	83	Glutathione peroxidase 3	8.26/25	5.99/23			1.40**^b^**	Protects cells and enzymes from oxidative damage
10	P04639	389	Apolipoprotein A-I	5.52/30	6.07/23	1.95^a^	2.08^a^	1.65^a^	Anti-inflammatory response, lipid metabolism
11	P14630	278	Apolipoprotein M	5.73/21	5.9/20	0.70**^b^**	0.71**^b^**	0.61^a^	lipoprotein metabolic process, antioxidant acitivity
12	P04916	159	Retinol-binding protein	5.69/23	6.9/20		0.66**^b^**	0.73**^c^**	Acute-phase response, lung development
13	P17475	368	Alpha-1-antiproteinase	5.70/46	5.06/58		1.48^a^	1.25**^c^**	Acute-phase response, inflammatory response, response to hypoxia
14	Q63041	494	Alpha-1-macroglobulin	6.46/168	5.6/39		2.04^a^	1.25**^c^**	Able to inhibit all four classes of proteinases by a unique ‘trapping’ mechanism
15	Q63041	435	Alpha-1-macroglobulin	6.46/168	5.72/37			1.32^b^	Able to inhibit all four classes of proteinases by a unique ‘trapping’ mechanism
16	P06866	191	Haptoglobin	6.10/39	5.73/35	1.23**^c^**		1.23**^c^**	Acute inflammatory response, response to hypoxia
17	P02650	380	Apolipoprotein E	5.23/35	5.35/34		0.76**^c^**	0.52^a^	Negative regulation of inflammatory response, exhibit antioxidant property
18	P02651	255	Apolipoprotein A-IV	5.12/44	5.25/43			0.71**^b^**	Activation of LCAT
19	P04276	568	Vitamin D-binding protein	5.65/55	5.75/55	2.11^a^	2.13^a^	1.33**^b^**	In plasma, it carries the vitamin D sterols and prevents polymerization of actin by binding its monomers
20	Q9QX79	400	Fetuin-B	6.71/42	5.91/60	1.21**^c^**		0.66**^b^**	Acute phase anti-inflammatory mediator
21	P26644	164	Beta-2-glycoprotein 1	8.59/34	6.98/57		0.56**^b^**		Plasminogen activation,
22	Q498E0	127	Thioredoxin domain containing protein 12	5.25/19	7.49/33	1.46**^b^**	1.43**^b^**	1.24**^c^**	Cell redox homeostasis
23	P17988	150	Sulfotransferase 1A1	6.37/34	7.27/32		1.60^a^	1.33**^b^**	Response to stress, regulation of blood pressure
24	P08649	101	Complement C4	6.99/193	7.3/30	0.55**^b^**		0.53^a^	Inflammatory response, complement activation
26	P19804	244	Nucleoside diphosphate kinase B	6.92/17	7.05/16	1.61^a^	1.89^a^	1.35**^b^**	Cellular response to oxidative stress, negative regulation of apoptotic process
28	P20767	188	Ig lambda-2 chain C	5.76/11	6.62/28			0.66**^b^**	Antigen binding
29	P20767	188	Ig lambda-2 chain C	5.76/11	6.52/29			0.76**^c^**	Antigen binding

a denotes P<0.001, ^b^demotes p<0.01 and ^c^denotes p<0.05.

### Validation of Differentially Expressed Proteins by Western Blot Analysis

**Figure 4:**
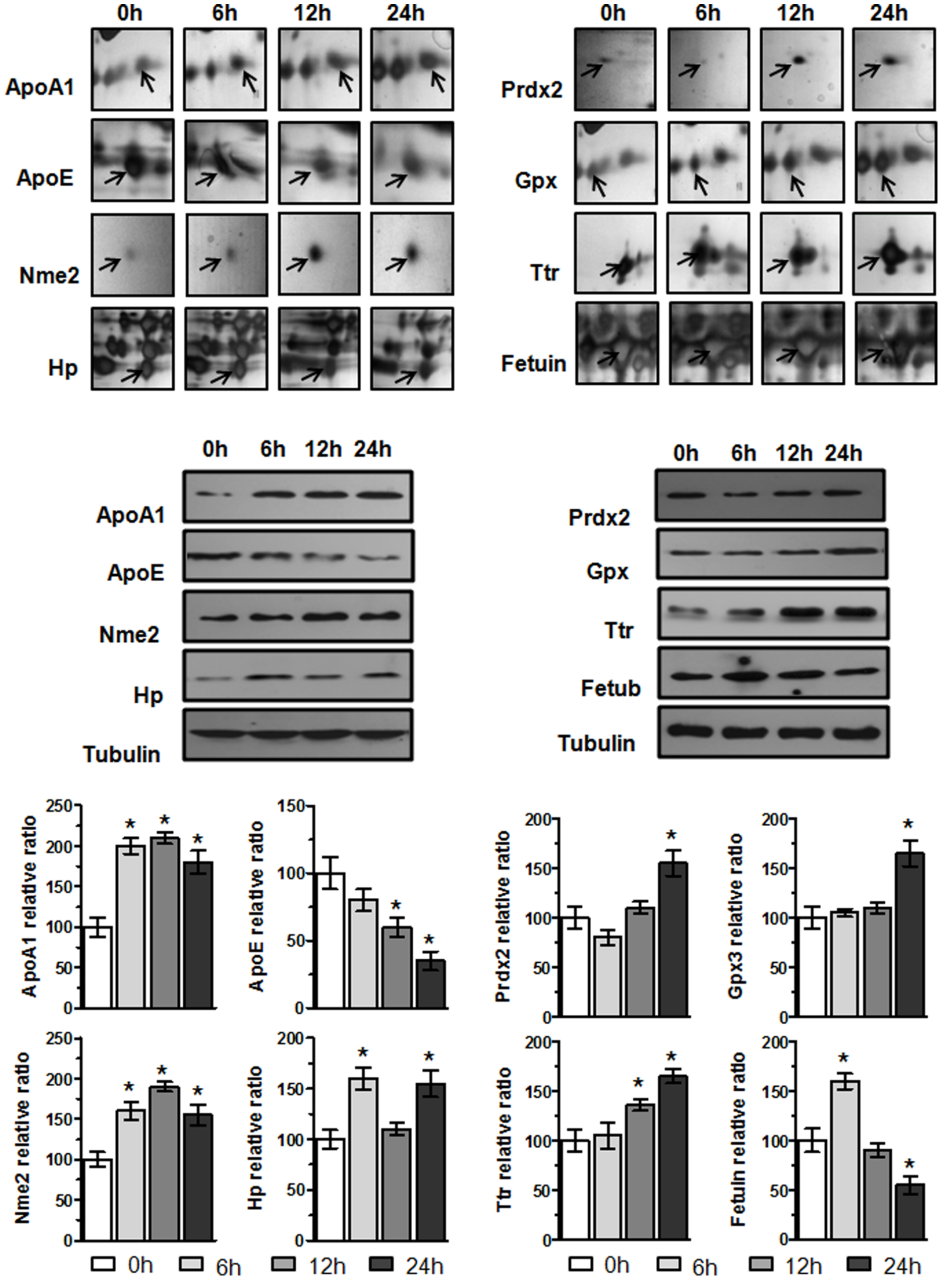
Validation of protein change patterns at different time points by Western blot analysis of proteins Ttr, Prdx-2, Gpx-3, Apo A -1, Hp, Apo-E, Fetub and Nme. Total plasma proteins (40 µg/lane) were separated by SDS-PAGE and probed with primary antibodies against these eight proteins.

We selected eight proteins Ttr, Prdx-2, Gpx-3, ApoA-I, Hp, Apo-E, Fetub and Nme, and further verified whether the expression patterns of proteins observed in 2-DE gels paralleled those validated by Western blot analysis. Band intensity was measured using Quantity One 1-D Analysis Software version 4.6.7 BIO-RAD, and the intensity ratio corresponding to β–tubulin band was calculated. Both Apo-E and Fetub were down-regulated in plasma after exposure to acute hypobaric hypoxia (Figure 4). The protein levels of Fetub increased gradually from 6 to 12 hr but down-regulated at 24 hr after acute hypobaric hypoxia. The expression of ApoA-I, Hp, Nme and Ttr increased gradually from 6 to 24 hr after acute hypoxia (Figure 4). Both Prxd-2 and Gpx-3 were up-regulated in plasma after acute hypobaric hypoxia and remained at high levels (Figure 4). The expression patterns of the selected proteins observed by Western blot were in agreement with 2-DE results, thus confirming the reliability of the proteomic analysis.

## Discussion

High altitude exposure depends not only on the speed of ascent, degree of hypoxia but also on the duration of stay at a given altitude. It is therefore, important to decipher the temporal proteomic changes in plasma for a better understanding of several altitude-induced pathophysiological mechanisms in order to identify new biomarkers or potential therapeutic targets. To our knowledge, this is the first comprehensive proteome study reporting the proteome level changes in plasma of rats exposed to acute hypobaric hypoxia. Our results obtained by 2-DE electrophoresis and partly also confirmed by the use of other techniques, indicate that short-term acute hypobaric hypoxia not only identify hypoxia-modulated early proteins but also altered distinct biological process depending on the stress duration. The relationships of these proteins with hypobaric hypoxia are elucidated in the following section.

### Regulation of Apolipoproteins in Plasma after Hypobaric Hypoxia

We found several apolipoproteins to be regulated in plasma of rat exposed to hypobaric hypoxia. These proteins function primarily as lipid binding proteins to transport lipids from the intestine to the liver and from the liver to tissues, including adipocytes, lung, heart, muscle, and breast tissues. In recent studies, apolipoproteins have been shown to have a clinical importance because of their roles in endothelial protection, anti-oxidation, and anti-inflammation [14], [15]. Here, we evidence the differential expression of apolipoproteins A-I, M, E, A-IV and H at different time points in plasma after acute hypobaric hypoxia. Specifically, apolipoprotein A-I (spot no.10) belong to the ApoA-I/A4/E protein family and is primarily produced in the liver and the intestine. ApoA-I can be found in the extracellular space and, being a structural component of high density lipoprotein (HDL), takes part in cholesterol absorption. ApoA-I up-regulation is associated with breast and lung cancer [16], [17]. Interestingly, studies provide new evidence supporting the notion that HDL plays a protective role in the lung. ABCA1, which interacts with lipid-poor ApoA-I, was earlier shown to be essential for maintaining normal lipid composition and architecture of the lung as well as respiratory physiology [18]. There is emerging evidence for the critical role of ApoA-I in protecting pulmonary artery and airway function as well as in preventing inflammation and collagen deposition in the lung [19]. More recently, proteomic studies revealed the anti-inflammatory role of ApoA-I in HAPE patients [20]. Here, we report ApoA-I expression increased gradually from 6 to 24 hr after acute hypoxia suggesting the anti-inflammatory role of ApoA-I. Spot 11 was identified as apolipoprotein M (Apo-M) which is produced in the liver and secreted in plasma. Apo-M is a member of the lipocalin family, a group of proteins with a characteristic coffee filter-like structure and a hydrophobic binding pocket [21]. It is a negative acute-phase protein that decreases during infection and inflammation [22]. Apo-M has also been shown to have antioxidant properties, protecting low density lipoprotein (LDL) against oxidative injury [23]. Plasma levels of Apo-M decreased gradually from 6 to 24 hr and the function of Apo-M was likely to be impaired during acute hypoxia, suggesting the decreased antioxidant activity of HDL. Apo-E (spot no. 17) is a mediator for binding, internalizing, and metabolism of lipoprotein particles. In addition to its role in lipoprotein metabolism, Apo-E may have other functions such as antioxidant [24], anti-platelet aggregation [25], anti-proliferative effects [26], and immunomodulation properties [27]. In this study, Apo-E was found to be down-regulated in plasma concentration after acute hypoxia. This could be related to the decreased antioxidant activity of Apo-E. Apolipoprotein A-IV (ApoA-IV, spot no. 18) is a glycoprotein that circulates freely or in association with chylomicrons and HDLs [31]. ApoA-IV has several proposed physiological roles, including lipid transport, lipoprotein metabolism, and control of food intake and gastric functions [28]. Most recently, the findings in studies of transgenic mice have supported a role for ApoA-IV in protection against atherosclerosis [29], [30]. Recent in vitro studies have supported an antioxidant action of Apo A-IV [31]. In this study, we found that ApoA-IV was down-regulated in the plasma after acute hypoxia. Additionally, apolipoprotein H also named as β-2 glycoprotein-1 (Apo-H, spot no.21) binds to various kinds of negatively charged substances, including heparin, phospholipids, and dextran sulfate. Apo-H may prevent activation of the intrinsic blood coagulation cascade by binding to phospholipids on the surface of damaged cells. Apo-H is synthesized in liver and secreted into the plasma. High levels of pathogenic Apo-H antibody in turn could cause hypercoagulation and venous and arterial thrombosis, and is clinically relevant to anti phospholipid syndrome [32]. Apo-H was found to be down-regulated in plasma after acute hypobaric hypoxia in this study.

### Regulation of Acute Phase Proteins in Plasma After Hypobaric Hypoxia

In general, concentrations of various positive and negative regulators of acute phase proteins (APP) increase or decrease in response to inflammation [33]. As observed in plasma of normobaric and hypobaric hypoxia exposed rats, six APP were regulated. We found enhanced levels of positive acute phase regulators, such that Alpha-1-antiproteinase and haptoglobin in plasma after acute hypobaric hypoxia. Alpha-1-antiproteinase (spot no.13) is a serine protease inhibitor (serpin) that functions as an antitrypsin as well as an antithrombin [34]. Its primary targets include elastase, plasmin and thrombin. A1AT protects the connective tissues (elastin) from inflammatory enzymes such as elastase in the lungs and pulmonary system, as well as helps to prevent blood coagulation. Haptoglobin (Hp, spot no. 16) is produced mainly in the liver and normally released in the blood during acute and chronic inflammation [35]. In addition to transporting hemoglobin to the liver, thus facilitating hemoglobin catabolism and preventing tissue injury, several functions have been assigned to Hp, including anti-oxidant activity, angiogenesis, and the host defense response to infection and inflammation [36]–[37]. It can be inferred that A1AT and Hp might play important role in defense in hypobaric hypoxia characterized by inflammation and accumulated hemoglobin. Tansthyretin (Ttr, spot no. 3, 4, 5), also called prealbumin, is a beta-sheet rich protein. It is synthesized predominantly by hepatic parenchymal cells. It behaves as a tetramer and binds to retinol binding protein (RBP) and thyroxin in plasma [38]. Because Ttr is a tryptophan-rich protein, the protein is used as a useful marker for nutrition assessment (NST). However, Ttr is also an anti-acute phase protein, and its levels are influenced by various conditions, such as inflammation and infection. A persistent low level of serum transthyretin is predictive of lethality, whereas increased levels were associated with improved ventilator performances. In this study, we found that Ttr was up-regulated in the plasma of rats exposed to hypobaric hypoxia. Alpha-1-macroglobulin (A1m, spot 14, 15) is a glycoprotein secreted in plasma. This protein presumably plays a role in the regulation of the immune system, and is involved in defending tissue against oxidation by reactive oxygen species and heme [39], [40]. A1m is known to be present in association with macromolecules (immunoglobulin A, albumin, and prothrombin), as well as in a free monomeric form protein [41]. This protein is considered to be a marker of renal insufficiency, and to reflect the overall inflammatory status in patients with arterial hypertension and normal renal function [42]. Recent studies have shown the medical use of alpha-1-microglobulin as an antioxidant in the treatment of diseases related to oxidative stress, infection, inflammation, ischemia-reperfusion, prophylaxis or conditions associated with the presences of free radicals and/or free haemoglobin [43]. In this study, the plasma level of A1m was up-regulated after acute hypobaric hypoxia. Another reported negative APP, fetuin (also called α-2-HS-glycoprotein) which was usually expressed at a decreased level in acute inflammatory condition caused by major surgical procedures, trauma, and burns [44], showed lower plasma levels in rat exposed to hypobaric hypoxia. Additionally, fetuin has been shown to function as an acute phase anti-inflammatory mediator that is critical to regulating the innate immune response following tissue injury. We also observed regulation of complement C4 (spot no. 24) which plays a central role in the activation of the classical pathway of the complement system. The complement system, composed of over 30 proteins that act in concert to protect the host against invading organisms, initiates inflammation and tissue injury [45]. Complement activation promotes chemotaxis of inflammatory cells and generates proteolytic fragments that enhance phagocytosis by neutrophils and monocytes. Collard et al. showed complement activation by endothelial cells in vitro in response to hypoxia/reoxygenation [46], providing a possible link between endothelial oxidant generation and neutrophil adhesion and activation. In this study, complement C4 was down-regulated in plasma of rat after acute hypobaric hypoxia. Retinol binding protein (RBP 4, spot no.12) is a vitamin-A transport protein that acts as an adipokine when secreted from adipose tissue. RBP 4 down-regulates GLUT4 [47], the insulin-activated glucose transporter responsible for translocation of glucose into both muscle and fat cells [48], and has also recently been shown to induce expression and secretion of pro-inflammatory cytokines in primary human macrophages known to induce insulin resistance [49]. Recent studies have demonstrated that during the onset of a stress reaction of medium severity, the reduction of RBP plasma levels by half releases more retinol in free form into the extracellular space in amounts corresponding to about 10 times the normal free concentration [50]. In this study, we also found that RBP was down-regulated after hypobaric hypoxia. The low plasma concentration of RBP might result from the declining synthesis of RBP in rat livers.

### Regulation of Binding Proteins in Plasma After Hypobaric Hypoxia

Vitamin D-binding protein (VDBP, spot no. 19) is a multifunctional protein found in body fluids and on the surface of many cell types. In plasma, it carries vitamin D sterols and prevents polymerization of actin by binding its monomers. VDBP also associates with membrane-bound immunoglobulin on the surface of B lymphocytes and with IgG Fc receptors on the membranes of T lymphocytes, suggesting its possible role in the immunopathogenesis and progression of the disease [51]. Besides serving as a transporter of vitamin D, VDBP also plays an important role in response to tissue injury, in which VDBP can be converted to a macrophage activating factor (VDBP-MAF) to stimulate macrophages [52]. Another role of VDBP in response to injury is to scavenge for vascular and extracellular actin as a result of cellular necrosis, and VDBP has been shown to be in lower circulating concentrations in the presence of inflammatory or necrotic diseases [51], [53]. Furthermore, VDBP is also implicated in a numerous diseases, including chronic obstructive pulmonary disease, cancer, and trauma [54]–[56]. In present study, we found that the plasma concentration of VDBP was increased significantly, suggesting the protective role in defense in hypobaric hypoxia.

### Role of Antioxidant and Oxidative Stress Associated Proteins during Hypoxia

In this study, three cellular antioxidants, thioredoxin domain containing protein 12, peroxiredoxin-2 and glutathione peroxidase 3 were identified in the plasma of rats treated with hypobaric hypoxia. Thioredoxin domain containing protein 12 (TXNDC12, spot no. 22) belongs to the thioredoxin super family. Members of this super family possess a thioredoxin fold with a consensus active-site sequence (CxxC) and have roles in redox regulation, defense against oxidative stress, refolding of disulfide-containing proteins, and regulation of transcription factors [57]. In this study, TXNDC12 was up-regulated, suggesting the protective of TXNDC in the cellular defense against oxidative stress caused by hypobaric hypoxia. Peroxiredoxins (PRDXS) are H_2_O_2_ scavenging antioxidant proteins and six mammalian isoforms (I–VI) have been identified. They are small proteins (22–27 kDa) and all have the common thioredoxin CxxC motif and catalyze peroxide detoxification [58]. Increased expression of different peroxiredoxins has been reported in lung cancer, alveolitis, and hypoxic conditions [59]. Peroxiredoxin-2 (PRDX2, spot no. 8) belongs to a ubiquitous PRX family, which has been shown to have multiple functions such as enhancing natural killer cell activity [60], increasing cell resistance to oxidative stress [61], regulating transcription activator protein [62], protecting erythrocytes against oxidative stress [63], and anti- HIV activity [64]. In light of its multifaceted biological functions, the increased plasma level of PRDX2 might play a multifunctional role during the acute inflammation induced by hypobaric hypoxia. Glutathione peroxidase 3 (GPX-3, spot no. 9) functions in response to oxidative damage by catalyzing the reduction of hydrogen peroxide, lipid peroxides, and organic hydroperoxide. Rousseau et al. reported the increase in plasma glutathione peroxidase activity as a potential indicator of hypoxic stress in breath-hold diving [65]. Interestingly recent study from our group showed that ‘hypoxia-sensitive’ animals fail to increase GPX3 after acute hypobaric hypoxia [66]. Despite significant difference in experimental conditions employed in these two studies (exposure time and altitude), it is likely that the upregulation of GPX-3 could play a role in acclimatization to hypobaric hypoxia. Sulfonate conjugation is an important pathway in the metabolism of a variety of endogenous and exogenous compounds, including estrogens and other mammary carcinogens. This reaction is catalyzed by SULTs, 3 a superfamily of multifunctional enzymes including six cytosolic SULTs that have been identified in human tissues. Sulfotransferase 1A1 (SULT 1A, spot no: 23) is one of the most important members in this enzyme family due to its extensive tissue distribution and abundance. This enzyme has a substantially higher activity than other SULTs in catalyzing the sulfonation of 4-nitrophenol, a commonly used assay in biochemical pharmacogenetic studies for testing the activity of thermostable phenol SULTs [67]. SULT1A1 catalyzes the sulfonation of estrogens to form water-soluble and biologically inactive estrogen sulfates, reducing the level of estrogen exposure in their target tissues [68]. The mutation in the SULT1A1 gene would affect an individual’s capacity to efficiently sulfate endogenous compounds, drugs and xenobiotics, and consequently result in an individual’s susceptibility to cancer [69]. Yunfei et al. reported SULT 1A1 polymorphism as a predisposition factor for lung cancer [70]. In this study, Sulfotransferase 1A1 was up-regulated in plasma of hypobaric hypoxia treated rats. Other proteins detected include nucleoside diphosphate kinase (NME, spot no: 26), a multifunctional enzyme involved in the maintenance of the cellular pools of nucleoside triphosphate and in transcriptional regulation [71]. Nucleoside diphosphate kinase has been shown to undergo S-thiolation or disulfide cross-linking under conditions of oxidative stress, which could have implications in function switching [71]–[73]. In this study, NME was up-regulated, suggesting the protective role against the oxidative stress induced by hypobaric hypoxia.

In this experiment, candidate proteins corresponding to spots 6, 7 and 27 could not be detected in the database. They may be novel proteins, or else they may be small fragments of some proteins, as can be suggested from their low molecular weight. The ESI-MS/MS study of these spots may lead us to identify these novel proteins.

### Study Limitations

We have noted some important limitations of our study. First limitation is that the protein quantification is based on only 2D-gels. Most of the proteins have multiple isoforms that differ in electrophoretic mobility. We could not estimate all the isoforms of the identical protein in 2D-gel, and this may affect the accuracy of the single protein quantification. Second limitation of the 2D gel-based proteomic approach is the variable validity of the protein identification. Some gel spots may contain more than one protein. The identification of proteins from peptide sequence is calculated as a high probability. Therefore, the validity of the approach has to be confirmed by different methods. However, since only a few of the respective antibodies against the observed proteins are currently available, it remains necessary to confirm the validity of our gel-based protein expression data by antibody-based techniques (e.g Western blotting), whenever antibodies become available. Third, although our work has provided some important clues, further studies are still needed to elucidate the detailed roles of these differentially expressed proteins during acute hypobaric hypoxia exposure. In addition to this, while we feel that altitude decompression sickness is unlikely, we cannot exclude the possibility that some of the findings may be related to the secondary effects of bubbles in tissues or blood. Exclusion of this possibility will require normobaric hypoxic exposures.

## Conclusions

The maintenance of oxygen homeostasis is crucial during hypobaric hypoxia and a better comprehension of the pathways involved in the response to changes in oxygen availability might have important biological and therapeutic implications. Cells can respond to changes in oxygen availability with a rapid feedback mediated through post-translational modifications or membrane depolarization and with a late hypoxic response pathway altering gene and protein expression over several hours. These last changes are mediated, at least in part, through the induction of hypoxia-inducible transcription factors as HIF which is considered a marker of the ability of the cells to respond to the hypoxic condition. Even though several genes, that are modulated by HIF, have been described in numerous cell lines undergoing hypoxia, a comprehensive temporal proteomic analysis of changes occurring in the plasma protein profile after acute hypobaric hypoxia is still absent. This study indicates that acute hypobaric hypoxia exposure modulates protein profile by inducing significant changes in the expression of atleast 25 proteins. The 25 identified proteins can be classified into different functional categories, according to the GO annotation system, indicating that hypoxia might interfere with a broad range of physiological and pathological functions including hypoxia response, inflammation, cellular response to oxidative stress and reactive oxygen species, homeostatic process, lipid metabolism and apoptosis. Although none of the proteins alone are univocally associated with hypobaric hypoxia, the combination of proteomic information of different proteins is significant to give a better understanding of the molecular pathway affected by hypobaric hypoxia. Further investigation will be addressed to assess the specific roles and functional correlation of these proteins as well as regulation of hypoxia in rats, widen the temporal window for therapeutic interventions, and introduce novel therapeutic targets.

## Methods

### Collection and Isolation of Plasma Samples

Male Adult male Sprague-Dawley rats (n = 36) weighing approximately 220±10 g were used for the study. Animals were maintained in the animal house facility of the Defence Institute of Physiology and Allied Science (DIPAS) with a 12-hr light/dark cycle and were provided with food and water at *ad libitum*. To study the effects of acute hypobaric hypoxia exposure, 36 male Sprague-Dawley rats (220±10 g) were randomly divided into four groups (I to IV; n = 9). Group I served as the normoxia group maintained in standard environment. Groups II, III and IV served as hypoxia groups where the rats were exposed to simulated hypobaric hypoxia for 6 h, 12 h and 24 h respectively, at 25,000 ft (7620 m, 282 mm Hg, 37.60 kPa) in a specially designed animal decompression chamber where altitude could be maintained by reducing the ambient barometric pressure and temperature and humidity could be precisely controlled. The temperature and humidity was maintained at 28±2°C and 60±5% respectively. The rate of ascent to altitude was maintained at the rate of 300 m/min and it took a period of 20–25 min to reach the desired altitude. Being a slow rate of ascent in altitude (gradual decrease in ambient pressure), it is less likely to induce decompression induced gas bubbles during exposure. All the experimental protocol and animal care was approved by the ethical committee of the Defence Institute of Physiology and Allied Sciences (27/1999/CPCSEA) in accordance with the guidelines of “Committee for the Purpose of Control and Supervision of Experiments on Animals” of Govt. of India. Blood was withdrawn from the ocular vein immediately after 6, 12 and 24 h hypoxia. To prepare plasma, anticoagulants (either EDTA, heparin or sodium citrate), were added to the blood samples immediately after the blood was drawn to prevent clotting (EDTA plasma: 10 mL containing approximately 1.7 mg potassium EDTA; heparin plasma: 5 mL containing heparin 1 vial; sodium citrate plasma: 10 mL containing 1 mL of 0.118 mol/L (3.2%) citrate solution). The specimens were then centrifuged at 1500 g for 10 min/4°C to avoid hemolysis, decanted and transferred into Eppendorf tubes as aliquots. To each 1.0 mL plasma aliquots, 10 µl of protease inhibitor were added to obtain the reproducible results by 2-DE analysis. Proteases used are less active at lower temperature; therefore it is recommended that the plasma be prepared at as low a temperature as possible. The plasma samples were stored at –80°C until further analysis. For optimal reproducibility, plasma proteins from each of the nine sample pairs of rats (hypoxia and controls) were processed together throughout the 2-DE procedure. The protein concentration in plasma was determined using Bradford reagent.

### Acetone/TCA Precipitation

A 100 µl of plasma sample was diluted with 900 µl of 10% TCA in acetone. The mixture was incubated overnight at−20°C and centrifuged at 15000 g, 4°C for 10 min. The supernatant was removed and 1000 µl of 90% ice-cold acetone were added to wash the pellet. The sample was incubated at −20°C for 10 min and centrifuged as above. The acetone containing supernatant was removed and the pellet was air dried. For 2-D gel electrophoresis, the protein pellet was suspended in 100 µl of lysis buffer as described earlier [74]. The protein sample was stored frozen at −20°C until analysis.

### First-dimensional IEF Using the Protean IEF Cell

Total protein content in plasma samples was determined by Bradford assay and employed bovine albumin standards. Immobilized linear pH gradient strips (17 cm, pH 5–8, Biorad ) were rehydrated with the individual plasma samples, 500 µg of protein, in 300 µl of a improved rehydration buffer solution as described earlier (13) containing 7 M urea, 2 M thiourea, 1.2%, w/v CHAPS, 0.4% w/v ABS-14, 20 mM dithiothreitol (DTT), 0.25%, v/v, pH 3–10 ampholytes and 0.005% w/v bromophenol blue (BPB), for 18 h without current (in-gel passive rehydration). After rehydration, the focusing tray was renewed to remove any proteins not absorbed into the strip. IEF was conducted using a Protean IEF Cell (Bio-Rad) at 20°C as follow: 250 V for 1 h (slow ramping), changing the wicks every 30 min (to assist removal of ionic contaminants), 1000V for 1 h, linear ramping 10 000 V to over 3 h and a constant of 10 000V until approximately 60 kVh was reached. Strips were removed and stored at –80°C until run on the second dimension.

### Second-dimensional Electrophoresis

For 2-DE analysis, individual samples from each animal group (Groups I-IV; n = 9) were run thrice (9×3 = 27 gels). Prior to SDS-PAGE, the IPG strips were equilibrated twice for 15 min with gentle shaking. The first equilibration solution contained 50 mM of Tris-HCl, pH 8.8, 6 M urea, 30% v/v glycerol, 2% w/v SDS, 1% w/v DTT and 0.01% w/v BPB. In the second equilibration solution, DTT was replaced with 2.5% (w/v) iodoacetamide. The equilibrated IPG strips were slightly rinsed with milli-Q water, blotted to remove excess equilibration buffer and then applied to SDS-PAGE gels (20 cm × 20 cm × 1 mm 8–19% polyacrylamide (30% (w/v ) acrylamide:0.8% (w/v) bis-acrylamide, 37.5∶1 stock) using a PROTEAN II XL system (Bio Rad) at 10 mA per gel for 30 min followed by 35 mA per gel for 12 h until the dye front had run off the edge of the 2-D gel.

### Staining and Imaging

After electrophoresis, proteins were visualized by modified silver staining procedure compatible with MS [75]. The gels were fixed in 50% v/v methanol, 12% v/v acetic acid and 0.05% v/v formaldehyde for at least 2 h. The fixed gels were rinsed with 50% v/v ethanol three times for 20 min each, then again sensitized with 0.02% w/v sodium thiosulfate followed by three washings with milli-Q water each for 20 s. The gels were immersed in 0.1% w/v silver nitrate and 0.075% v/v formaldehyde for 20 min and rinsed with milli-Q water twice for 20 s each. It was developed with 6% sodium carbonate and 0.05% v/v formaldehyde. Finally, the reaction was terminated by fixing with 50% v/v methanol and 12% v/v acetic acid. The stained gels were imaged using an Investigator ProPic II Genomics Solutions. Gel images were exported as 16-bit-gray scale TIFF files that were imported into 2Dprogenesis analysis software package. The principles of measuring intensity values by 2D analysis software are similar to those of densitometric measurement. After the background subtraction the protein spots were automatically defined and quantified with the feature detection algorithm. Spot intensities were expressed as relative volumes in percentages (% volume) by integrating the OD of each pixel in the spot area (vol) and dividing with the sum volumes of all spots detected in the gel. A master gel was prepared for each group, after considering one gel from each animal of the group, using Progenesis Software, thus representing plasma proteome of each individual group. In the master gel each spot is assigned a unique number. The quality of the match made by the computer was critically evaluated in each case, and necessary editions and corrections were done manually. Initially, protein spots with significant changes test (p≤0.05, one –way ANOVA) in a consistent direction (increase or decrease) were cut for identification, according to the method of Turko et al. [76]. Mostly, only spots that are statistically different between control and experimental groups were subjected to in-gel trypsin digestion for subsequent analysis by mass spectrometery.

### MS identification of Proteins

#### In-gel digestion with trypsin and extraction of peptides

The procedure for in-gel digestion of protein spots from silver stained gels was performed. In brief, protein spots were extensively washed with ultrapure water and each gel spot was excised with a clean scalpel. The spots were destained and incubated for 30 min with 30 mM potassium ferricyanide and 100 mM sodium thiosulfate at room temperature. The gel pieces were rinsed several times with water to remove destaining solution. The gel pieces were washed for 15 min at room temperature with water and 50 mM NH_4_HCO_3_/Acetonitrile. Enough acetonitrile were added to cover gel pieces for shrinking the gel pieces. The gel pieces were rehydrated in 10 mM NH_4_HCO_3_ for 5 min, equal volume of acetonitrile were added and removed after 15 min of incubation. The gel pieces were again covered with acetonitrile and removed. The gel pieces were dried in a vacuum centrifuge. The dried gel pieces were digested with 20 µl of trypsin (20 ng/µl, Trypsin Singles Proteomics Grade, Sigma) and incubated the sample at 37°C overnight, the tryptic peptide were sonicated for 10 min and dried in a speed Vac. The dried peptides were extracted with 5 µl of 0.1% TFA.

#### MALDI-TOF/TOF

For PMF, in-gel tryptic peptides of each spot of interest were mixed with an acidic solid matrix such as α-cyano-4-hydroxy cinnamic acid (CHCA) matrix 10 mg/ml, which provides high sensitivity and negligible matrix adduction during the laser absorption and subjected to laser radiation. The matrix was made in 70% acetonitrile and 0.03% TFA. 0.5 µl of the peptide extracts mixed with the 0.5 µl of the matrix were manually spotted onto a 600 µm/384 well AnchorChip sample target (Bruker Daltonics) and dried at ambient temperature. Peptide mass spectra were recorded in the reflectron mode using an Ultraflex III Tof/Tof mass spectrometer (Bruker Daltonics) equipped with a 384-sample scout source. The ion acceleration voltage after pulsed extraction was 29000 V. A peptide calibration standard (Bruker Daltonics) was used for external calibration as previously described [20]. MS and MS/MS data were recorded automatically on the MALDI-TOF/TOF instrument using the three most abundant peptide signals of the corresponding peptide mass fingerprint (PMF) spectrum. The monoisotopic peak list was generated in Post Processing s/w and True peptide mass list was generated by Bruker Flex Analysis software version 3.0 and Biotools ver 3.1 without using the smoothing function and the peak filter was applied to exclude the masses lower than 700 Da and the signal to noise ratio of 20. The generated peptide mass list was searched with MASCOT (http://www.matrixscience.com) using entire Uniprot/Swiss-Prot protein database to find and match the protein identity. Databases searches were performed by using following search paprmetes; *Rattus norvegicus* as taxonomy, carbamidomethyl modification of cysteines and possible oxidation of methionine, one missed cleavage, a mass accuracy of ≤100 ppm was requested for PMF and for MS/MS searches, a mass accuracy of ≤70 ppm was allowed for peptide masses and their fragments, respectively. Before mass analysis, a further confirmation was made if the differentially expressed proteins were present in at least five gels out of 9 gels from each of the four respective groups (n = 9) used for preparing the master gel. For each identified Protein, at least one Peptide was selected for MS/MS (TOF/TOF) to validate the Protein Identity. Instrument was used in the Lift mode (TOF/TOF) to obtain the MS/MS spectra. Again the Flex Analysis3.0 and Biotools 3.1 s/w were used to generate the fragments mass list and the sequence Tag of peptide. The mass list was sent to database in same way as was done in case of above PMF approach. The mass tolerance error of 0.5 Da to 1.0 Da was used for MS/MS ion search. The MS/MS ion search confirmed the protein identity and provided the amino acid sequence of particular peptide. Gene ontology (GO) annotations (functional distribution) for identified proteins were assigned using Blast2GO research tool [77].

### Western Blot

The protein quantification of transthyretin, peroxiredoxin-2, glutathione peroxidise, Apo A-I, haptoglobin, Apo-E, fetuin–B and Nucleoside diphosphate kinase B were selected to be validated by Western-blot analysis because the expression changes of these proteins were more obvious than that of the other proteins and the obtaining of their antibodies was convenient. Briefly, plasma samples were first diluted 10 times by 1 X PBS, and then total proteins (40 µg) were separated by SDS-PAGE and electro-blotted to nitrocellulose membrane. After being blotted with 5% nonfat-dried milk in 1 X TBST (25 mM Tris, pH 7.5, 150 mM NaCl, 0.1% Tween 20) overnight, membranes were incubated with primary antibodies for 2 h, followed by secondary antibody for another hour. All these experiments were conducted at room temperature. The immunocomplexes were visualized by chemiluminescence using the chemiluminescent peroxidase substrate kit (Sigma-Aldrich, St. Louis Mo 63103, USA). The quantification of protein was done by densitometric digital analysis of protein bands (TIFF image) using Quantity One 1-D Analysis Software version 4.6.7 (BIO-RAD, Hercules, CA). Each protein band was normalized to the corresponding β–tubulin band. The results of western blot are representations of three independent experiments (Mean ± SEM). Statistical analyses were performed using GraphPad Prism (Version 5) one- way ANOVA with pair-wise multiple comparison procedures (student-Newman-keuls method ), and a p-value of <0.05 was considered significant.
